# Remission of type 2 diabetes: always more questions, but enough answers for action

**DOI:** 10.1007/s00125-023-06069-1

**Published:** 2024-01-08

**Authors:** Amy Rothberg, Michael Lean, Blandine Laferrère

**Affiliations:** 1 Division of Metabolism, Endocrinology & Diabetes, University of Michigan, Ann Arbor, MI USA; 2 School of Medicine, Dentistry and Nursing, College of Medical, Veterinary and Life Sciences, University of Glasgow, Glasgow, UK; 3 Division of Endocrinology, Columbia University Irving Medical Center, New York, NY USA

**Keywords:** Bariatric surgery, Beta cell reserve, Calorie restriction, Cost-effectiveness, Glucagon-like peptide-1, Relapse, Remission, Review, Weight loss

## Abstract

The concept of type 2 diabetes remission is evolving rapidly, and gaining wide public and professional interest, following demonstration that with substantial intentional weight loss almost nine in ten people with type 2 diabetes can reduce their HbA_1c_ level below the diagnostic criterion (48 mmol/mol [6.5%]) without glucose-lowering medications, and improve all features of the metabolic syndrome. Pursuing nomoglycaemia with older drugs was dangerous because of the risk of side effects and hypoglycaemia, so the conventional treatment target was an HbA_1c_ concentration of 53 mmol/mol (7%), meaning that diabetes was still present and allowing disease progression. Newer agents may achieve a normal HbA_1c_ safely and, by analogy with treatments that send cancers or inflammatory diseases into remission, this might also be considered remission. However, although modern glucagon-like peptide-1 receptor agonists and related medications are highly effective for weight loss and glycaemic improvement, and generally safe, many people do not want to take drugs indefinitely, and their cost means that they are not available across much of the world. Therefore, there are strong reasons to explore and research dietary approaches for the treatment of type 2 diabetes. All interventions that achieve sustained weight loss of >10–15 kg improve HbA_1c_, potentially resulting in remission if sufficient beta cell capacity can be preserved or restored, which occurs with loss of the ectopic fat in liver and pancreas that is found with type 2 diabetes. Remission is most likely with type 2 diabetes of short duration, lower HbA_1c_ and a low requirement for glucose-lowering medications. Relapse is likely with weight regain and among those with a poor beta cell reserve. On current evidence, effective weight management should be provided to all people with type 2 diabetes as soon as possible after diagnosis (or even earlier, at the stage of prediabetes, defined in Europe, Australasia, Canada [and most of the world] as ≥42 and <48 mmol/mol [≥6.0 and <6.5%], and in the USA as HbA1c ≥39 and <48 mmol/mol [≥5.7 and <6.5%]). Raising awareness among people with type 2 diabetes and their healthcare providers that remission is possible will enable earlier intervention. Weight loss of >10 kg and remission lasting 1–2 years may also delay vascular complications, although more evidence is needed. The greatest challenge for research is to improve long-term weight loss maintenance, defining cost-effective approaches tailored to the preferences and needs of people living with type 2 diabetes.

**Graphical Abstract:**

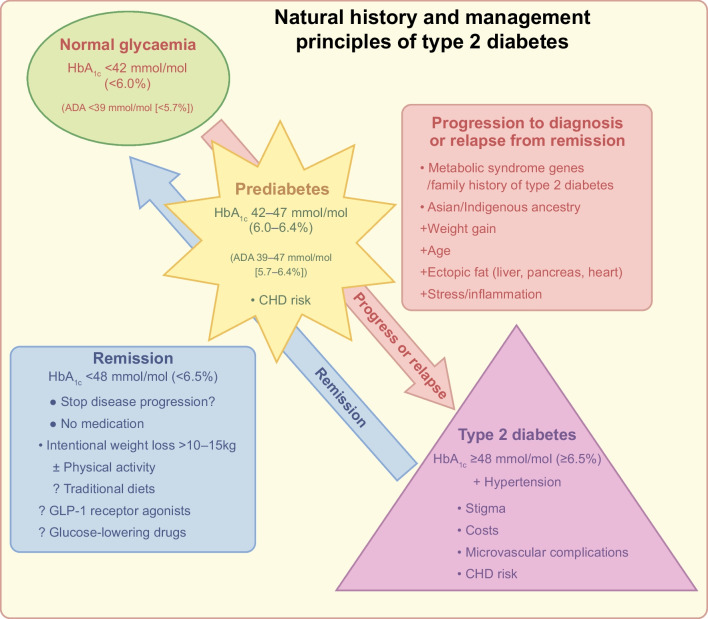

**Supplementary Information:**

The online version contains a slide of the figure for download available at 10.1007/s00125-023-06069-1.

## Introduction

We have made many advances over the last few years in the treatment of type 2 diabetes, with most focused on drugs to lower HbA_1c_ and reduce cardiovascular risk. However, multiple painful, disabling and life-shortening complications remain common. The concept of ‘remission’ was first introduced in diabetes care in the 21st century and is rapidly becoming embedded into routine clinical practice. However, its implications need exploration and re-thinking, both for people living with diabetes and for health professionals. Central to the concept of remission is the distinction between being ‘free of the disease’ (i.e. not satisfying the diagnostic criteria) and having ‘freedom from the disease’ (i.e. the personal impacts of the disease and its treatments).

## Understanding remission

‘Remission’ means not having an active disease, with the implication that neither symptoms nor new complications should develop. Remission is a radical new concept for most people living with type 2 diabetes, and exciting but potentially unsettling for their doctors, most of whom believed that type 2 diabetes was a permanent, inevitably progressive disease, and whose training and experience with weight management is very limited. The term ‘remission’ is familiar in the context of cancer and relapsing–remitting inflammatory conditions. It implies that the disease process has been arrested but can be reactivated, resulting in relapse, and is distinct from a ‘cure’, in which an entire disease process is eradicated.

In some other fields, spontaneous disease remission may occur, but achieving remission is often a therapeutic triumph, often demanding highly invasive treatments with risks of serious complications and often a need to take medications indefinitely to suppress the disease activity. However, most people dislike being ‘diseased’ and having to take medications and, indeed, in the case of type 2 diabetes, adherence to glucose-lowering medications is notoriously low [1].

Achieving type 2 diabetes remission requires substantial and sustained weight loss, inevitably demanding dietary restrictions. Historically, diet therapy has been accorded a very low status, with often just a passing mention in guidelines and clinical practice, and has attracted orders of magnitude lower research funding than other treatments. In reality, however, despite drug treatments, type 2 diabetes shortens lives to a similar degree as some major cancers [2–4], and still generates disabling, life-shortening complications. This is despite the fact that it is well known that effective weight management can improve glycaemia and all the associated features of the metabolic syndrome.

## Definition of type 2 diabetes remission

An international panel of experts defined remission of type 2 diabetes as an HbA_1c_ below the diagnostic threshold of 48 mmol/mol [6.5%] without taking glucose-lowering medication [5]. As type 2 diabetes is strongly age-related, the panel agreed that HbA_1c_ should be tested at least annually to confirm continued remission and that renal function be checked and a retinal examination be carried out regularly, as the pre-remission period of diabetes may already have initiated complications that may progress. Furthermore, as other features of the metabolic syndrome commonly coexist, it remains important to monitor and manage CVD risk factors after remission.

Some earlier publications have proposed a lower HbA_1c_ cut-off of 42 mmol/mol (6%) for remission of type 2 diabetes, that is, remission of prediabetes. The lower HbA_1c_ criterion used to define prediabetes is 42 mmol/mol (6.0%) by diabetes agencies in Europe, Australasia and Canada, but 39 mmol/mol (5.7%) by US agencies. This would be a stringent but worthy goal, as macrovascular complications already occur in the presence of prediabetes. Others have suggested that the term ‘remission’ could be applied to diabetes that is tightly managed with medication to achieve an HbA_1c_ <48 mmol/mol (<6.5%). While this might be expected to reduce the risks of microvascular complications, it presents unacceptable risks of hypoglycaemia if achieved with insulin or sulfonylureas, and excess deaths have been reported in clinical trials of medications pursuing near-normoglycaemia [6]. The newer glucagon-like peptide-1 (GLP-1) receptor agonists and sodium–glucose cotransporter-2 (SGLT2) inhibitors, in combination with good dietary support, have potential to enable more people with type 2 diabetes to achieve weight loss and/or normoglycaemia while avoiding hypoglycaemia, with renoprotection and decreased risks of CVD and mortality [7]. However, lifelong drug treatment is costly, impacts quality of life and is often not what people living with type 2 diabetes want [8]. Unfortunately, lifelong dietary restrictions, as currently presented, seem to be similarly unacceptable or unsustainable for many, as the benefits for what is largely asymptomatic early disease are perceived to be low.

## Determinants of remission and relapse

Weight loss and beta cell function are the main inter-related determinants of diabetes remission. The DiRECT randomised trial, which included 298 people with type 2 diabetes of up to 6 years’ duration, reported remission in the dietary intervention group of 46% at year 1, driven mainly by weight loss [9]. Remission declined with weight regain to 36% at year 2, and restoration of beta cell function (maximal insulin secretion), associated with loss of ectopic fat in the liver and pancreas, was demonstrated in individuals in remission at 2 years. Similarly, the DIADEM-1 randomised trial in Qatar found that weight loss of 10 kg resulted in 61% remission among 158 randomised participants with a mean diabetes duration of 21 months [10]. While the great majority of participants with ≥15 kg weight loss in DiRECT achieved remission for 2 years, a 14% minority of participants who lost >15 kg had no remission [9]. This may be because these participants did not lose enough weight; however, a similar percentage do not go into remission after bariatric surgery, revealing the presence of ‘non-weight loss-responsive type 2 diabetes’, in which there is loss of beta cell capacity resulting from long-term diabetes and/or conditions such as chronic pancreatitis. Other rarer types of diabetes, such as MODY and latent autoimmune diabetes in adults, may potentially be revealed by failure to achieve remission with weight loss. However, achieving substantial weight loss solely by dietary restriction is not easy and is even more difficult to maintain. Bariatric surgery typically results in greater weight loss (20–30%) that is better sustained over time [11] and associated with more prolonged remission of diabetes [12]. Both observational studies [11, 13] and RCTs [14] suggest that diabetes remission can occur even with a BMI >30 kg/m^2^ and may persist for many years. However, data vary, highlighting the importance of long-term studies [15].

While trials have shown that beta cell function can be restored by substantial weight loss, the beta cell reserve, or its clinical indicators, is a strong predictor of glycaemic improvement and remission after dietary weight loss [16, 17], after dietary intervention without weight loss [18] and after surgical weight loss [19–24]. Clinical indicators of better beta cell function and an increased chance of remission are lower HbA_1c_, the use of fewer medications, no requirement for insulin or sulfonylureas, a shorter known diabetes duration (often self-reported and not always reliable) and younger age. While type 2 diabetes relapse after gastric bypass surgery is associated with weight regain, the best predictor of relapse is poor presurgical beta cell function [25–27]. In one study, people with a known diabetes duration of <4 years had a 91% remission rate after surgery, with only 20% relapsing at 10 years; however, among those with a diabetes duration of ≥4 years only 41% achieved remission and 94% relapsed at 10 years. Initial weight loss impacted the immediate remission of type 2 diabetes but had only a marginal role in long-term relapse [28]. In another study including 2090 individuals in remission 2 years after gastric bypass surgery, the 20% who relapsed at 9 years were more likely to have been on insulin, have had an elevated HbA_1c_ and have had diabetes of longer duration prior to intervention [27]. In the DIBASY study, 37.5% of participants remained in remission 10 years after bariatric surgery compared with 5.5% of those receiving non-surgical management. There were more serious adverse events but fewer diabetes complications in those randomised to bariatric surgery. While the amount of weight lost was large and the dominant driver of remission, variations in weight changes did not predict diabetes remission or relapse within the surgical groups, probably because most participants greatly exceeded the necessary weight loss threshold. Weight regain at 10 years was similar among participants who sustained remission (7.1±6.9%) and those who had a diabetes relapse (8.2±6.2%) [29].

Therefore, while energy restriction and substantial weight loss are primary factors in inducing and maintaining type 2 diabetes remission, having a beta cell reserve capable of functional reactivation is an absolute necessity. Variability in relapse rates and duration of remission are likely to be explained by the same factors that influence the development and remission of type 2 diabetes: beta cell reserve and function, hepatic glucose output, and skeletal muscle mass and glucose oxidation. Aggravating influences come from the complex, inevitable effects of increasing age and of body fat reaccumulation or fat redistribution into ectopic sites. Both may be mediated in part by physical inactivity. Studies over many years are essential, as remission rates decrease over time and vascular complications take time to appear.

## Can remission be achieved without weight loss?

There is little evidence from the randomised trials that the macronutrient content of the diet affects weight control or remission, and weight loss remains the dominant factor behind remission in observational studies of, for example, low carbohydrate diets [30]. Other factors known to affect diabetes onset or control will have additional or modifying effects. The PREDIMED trial has shown that adopting a Mediterranean diet can prevent some new type 2 diabetes diagnoses without weight loss [31], which might point to a helpful approach for preventing relapse after remission. Intensive insulin therapy may provide a temporary period of remission [32–34], probably because of decreased lipotoxicity, glucotoxicity and inflammation and perhaps because it allows some beta cell redifferentiation [35]. GLP-1 receptor agonists provide weight loss with enhancement of beta cell function and glucose control, but the effect is rapidly lost on cessation of medication [36–38].

## Could remission reduce diabetes-related micro- and macrovascular complications?

The major micro- and macrovascular complications in diabetes are related to glycaemia. The diagnostic threshold for diabetes is set at the level at which microvascular complications specific to diabetes appear; they are very rare at HbA_1c_ <48 mmol/mol (<6.5%), so remission would be expected to reduce the risk of such complications. The very long time course of their development makes it difficult to establish prevention using an experimental RCT design. Instead, robust, long-term observational data are needed, for example from national registries. Single observational studies cannot prove causality, but the existing early evidence all supports the hypothesis that remission reduces or delays clinical complications of type 2 diabetes. In the Da Qing trial of a lifestyle intervention in people with prediabetes (*n*=~500), follow-up over 30 years showed persistent prevention of diabetes and suggested some reduction in cardiac complications [39]. In the Diabetes Prevention Program (DPP) (*n*=~3000), an intensive lifestyle intervention decreased CVD risk factors [40] and there was less microvascular disease in those who did not develop diabetes [41]. The intensive lifestyle intervention in the Look AHEAD trial, including >5000 participants with a mean type 2 diabetes duration of 6.8 years, resulted in a reduction in nephropathy and retinopathy [42] and in neuropathic symptoms [43]. Neither the DPP [44] nor the Look AHEAD trial [45] found that a sustained intensive lifestyle intervention, with variable and relatively small weight losses, decreased CVD events overall. However, in the Look AHEAD trial, in which the intensive lifestyle intervention resulted in remission for only 11.5% of participants at 1 year, declining to 7% at 4 years [46], a post hoc analysis found that cardiovascular events were reduced by 21% among participants who lost >10% of their body weight in the first year, and by 40% in those who achieved remission, an effect that appeared to be modulated by changes in weight, HDL-cholesterol and fitness [47]. Similarly, the 5-year follow-up of the DiRECT trial also found fewer clinical events after successful intervention.

The magnitude and sustainability of weight loss was also the key for remission and reduction in CVD complications in long-term bariatric surgery studies [12]. In the Swedish Obese Subjects trial, diabetes remission after bariatric surgery (72.3% at 2 years, 30.4% at 15 years) was associated with fewer macro- and microvascular complications over a median follow-up of 18 years [13]. Cohort analysis showed 47% fewer microvascular complications in individuals with type 2 diabetes 5 years after surgery (*n*=1111) compared with matched control participants who did not undergo surgery (*n*=1074) [48]. Other studies, supporting the trial evidence from Mingrone et al [29], have shown less progression of retinopathy at 5.9 years in a small cohort of individuals experiencing type 2 diabetes remission after bariatric surgery compared with those not in remission after surgery [49], and fewer CVD events (mainly cardiac failure) 10 years after surgery than in a well-matched cohort who did not undergo surgery [50].

The evidence is still limited but the pattern is consistent across studies of different designs: the risk of long-term complications of diabetes is likely to be reduced if remission can be achieved. The magnitude of weight loss and its sustainability are key, not only to normalise glycaemia, but also to reduce blood pressure and lipid levels. It would be valuable to identify the 15% of people with obesity and type 2 diabetes who do not achieve remission despite losing >15% of their body weight, to be able to provide a more personalised approach for targeted preventive and therapeutic risk reduction. This is not yet possible using currently available cardiometabolic risk clusters [51].

## Remission off medication vs equal diabetes control on sustained treatment

Challenging the internationally agreed definition of type 2 diabetes remission [5], it can be argued that HbA_1c_ <48 mmol/mol (<6.5%) might be achieved and maintained with sustained medication, that is, achieving freedom of the disease, but not necessarily freedom from the disease, with continued dependency on medication. There is a distinction here between diabetes control and glycaemic control, as substantial weight loss generating remission improves all features of the metabolic syndrome included within overall diabetes control. The emerging observational evidence suggests a delay in or prevention of diabetes complications after dietary weight loss that achieves remission [52, 53]. However, it has not been established whether achieving the same control of glycaemia, blood pressure and lipid levels using multiple medications rather than through weight loss by diet alone has the same clinical benefits. Newer potent diabetes medications such as GLP-1 receptor agonists and SGLT2 inhibitors, in addition to promoting weight loss, do provide some cardiovascular protection and renoprotection and a decreased risk of mortality, without the risk of hypoglycaemia [7]. These medications need to be tested with or against effective weight management.

## Precision medicine/information technology: one size does not fit all

As management strategies evolve to match new evidence, treatment needs to be personalised to meet individual needs, which change over time. Decisions must consider cost, quality of life and prediction models of remission outcomes, type 2 diabetes complications and cardiovascular risk. Obesity and type 2 diabetes are multifactorial diseases with many phenotypes [54]. Genetic testing can identify those with rare monogenic diabetes or MODY who need specific drug treatments. Subgroups of type 2 diabetes with different profiles of likely mechanisms and clinical outcomes have been identified in an effort to help individualise treatment and prevent diabetes complications [55], some of which may respond differently to bariatric surgery [56]. However, based on the present evidence, as a first step in a personalised approach, the most important subgroup of type 2 diabetes to identify is that of weight loss-responsive type 2 diabetes.

Before beginning treatment, it is important to make a full clinical and biochemical evaluation of risks and to discuss with the person living with diabetes and their family members or caregivers what these mean and the impact that different treatments (or no treatment) would have. We should be willing to discuss prognosis openly, just as we are with cancer patients; type 2 diabetes is a much more serious disease than many believe, so remission has real value. Before trying to give dietary advice, we must understand how powerful the human appetite is and how strong our social patterning of habitual behaviours is, and recognise that to sustain change some people need a lot more support than others.

## Remission is here to stay: what lies outside the box?

**Fig. 1:**
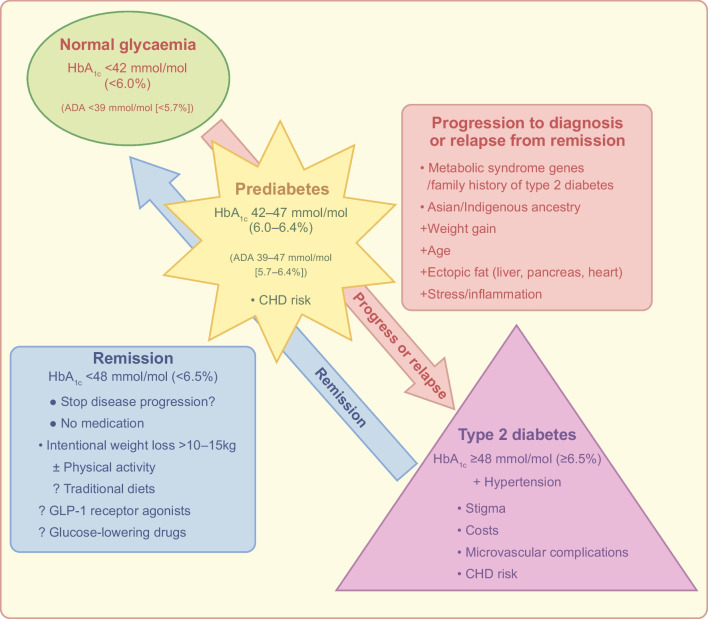
A schematic view of the progression of prediabetes to type 2 diabetes, in association with older age, weight gain, ectopic fat accumulation and stress/inflammation (pink arrow). Research has shown that lifestyle and weight management can prevent or even reverse progression, possibly resulting in type 2 diabetes remission (blue arrow). It should be noted that HbA_1c_ has an increasing association with CHD across its range. The lower HbA_1c_ criterion used to define prediabetes is 42 mmol/mol (6.0%) by diabetes agencies in Europe, Australasia and Canada, but 39 mmol/mol (5.7%) by US agencies. This figure is available as a downloadable slide

As discussed above, evidence has accrued over recent years that has changed our understanding of type 2 diabetes and pointed to a shift in emphasis in management, summarised in Fig. 1. Practical issues and questions remain. Do the metrics currently employed to define type 2 diabetes remission provide clarity, accuracy and recognition of the true state of diabetes? Where should research priorities lie?

From the evidence discussed, doctors must now revise the prevailing view of type 2 diabetes and provide proportionate evidence-based treatment and support. It should be noted that (1) type 2 diabetes is a very serious disease; (2) it arises from beta cell defects and excess body fat with ectopic fat accumulation; (3) it can be reliably sent into remission for at least 1–5 years by maintained weight loss of >10 kg; and (4) the evidence suggests that the disabling, painful and irreversible complications of type 2 diabetes are likely to be delayed or prevented by sustained remission. Weight management should therefore feature much more prominently in the management of type 2 diabetes than has been the rule. For example the 2022 ADA/EASD statement on managing hyperglycaemia in type 2 diabetes is still heavily dominated by recommendations for drugs and includes one page (out of 29) and 15 references (out of 346) on diet and weight management [57].

In a rapidly changing field, we are beginning to envision, and even to detail, an entirely new type of public health and diabetes care service, with remission as the number one management target for type 2 diabetes, aiming to minimise long-term medical consequences and costs to both patients and healthcare services. This should, in principle, be very attractive and potentially achievable, but many doctors and medical services view prescribing drugs as more expedient. Effective dietary treatment and/or GLP-1 receptor-based agonists [58] may be as potent as other medications for type 2 diabetes (and hypertension). Dietitians, or even lay people with specific training, might also have a greater impact on future health and life expectancy than doctors and drugs.

The evidence that weight loss and remission will not be sustained by all [59], or indefinitely, does justify the use of medications or surgery in appropriately selected cases, with the aim of reducing medical complications and enhancing health and well-being; however, medications and surgery are not alternatives of equal value. We have learned that treating one chronic disease, type 2 diabetes, however effectively, seldom has the global benefit we would like, because it leaves the underlying disease process of obesity to drive the other diseases it mediates [60]. Achieving and sustaining substantial weight loss is undeniably difficult in the post-industrial world, but the problems around prevention and secondary prevention of obesity and type 2 diabetes are not completely insurmountable for all if society decides to put genuine health promotion into action. We should do more than just reflect on the fact that pre-industrial traditional diets and lifestyles were associated with freedom from type 2 diabetes and related chronic diseases.

One emerging issue is the need to embrace a wider range of study designs, or determine the optimal study design, for research studies. For example, an RCT is often not the best study design to establish effect size once efficacy and safety (its principal purpose) are confidently demonstrated. When there is no interest in the possibility of a negative outcome (e.g. weight loss aggravating diabetes control), two-tailed statistical significance testing is irrelevant and results in unnecessarily large and expensive trials being conducted. This consideration does not apply to safety or clinical outcomes, but for that purpose mounting very long-term RCTs is impossibly expensive for a diet intervention, and a design depriving a control group of a proven treatment is ethically problematic. High-quality long-term observational studies in real-life settings are needed, on the clinical outcomes both of weight loss, achieved using different approaches, and of glycaemic control using glucose-lowering medications, including newer drugs with excellent CVD outcomes. Abandoning effective conventional treatment to pursue remission may not always be beneficial. For example, is the patient who takes metformin monotherapy and who maintains an HbA_1c_ of 41 mmol/mol (5.9%) less ‘free from disease’ than one with an HbA_1c_ of 46 mmol/mol (6.4%) but who takes no glucose-lowering medication? Both might avoid some downstream consequences of the disease. Pursuit of remission is most likely to be successful in the first few years after diagnosis or, even better, at the prediabetes stage [59]. Delaying pharmacotherapy that affords greater or easier weight loss, better diabetes control and potentially reduced clinical risks in an attempt to achieve drug-free remission may not be appropriate for everyone, nor even ethical.

## Patient and public involvement: well-being is key

Clinical research has too often assumed what is best for patients and failed to capture the lived experience of those living with type 2 diabetes: lower HbA_1c_ will not necessarily improve patient-reported outcomes. For example, the feasibility and acceptability of a structured, professionally supported dietary approach, with sustained improved well-being, were shown in the DiRECT trial [61, 62]. Individuals with type 2 diabetes have impaired health-related quality of life, which worsens further with additional comorbidities and complications, and more than a third have clinically important diabetes distress, related to the demands of self-management and the fear of complications. However, poorer glycaemic control is associated with higher diabetes distress scores and improvement in glycaemic control decreases diabetes distress and improves quality of life [63, 64].

The first ever position statement from the ADA on psychosocial care for people with diabetes recognised and recommended that diabetes care should prioritise health outcomes as well as quality of life and well-being [65]. Improved well-being should be the primary goal of all health interventions. Even in the wealthiest of countries, the proportion of people achieving glycaemic targets is low. Moreover, even though our strategies have evolved and can now result in type 2 diabetes remission, many people are not accessing diabetes prevention, control or remission, which should be the cornerstone of the therapeutic approach. Perhaps the debates around obesity and weight management are teaching us that the goals of diabetes management should embrace ‘better living’ for those with type 2 diabetes, starting with the need to optimise well-being, such as by providing safe and affordable housing and food, clean drinking water, a living wage, community support and childcare. We have a duty to lead the way in research towards better diabetes care, but that should be equitable within and between countries and cultures. Given that type 2 diabetes is most prevalent and increasing most rapidly in Asian and Indigenous populations, among people for whom modern healthcare and medications are neither accessible nor affordable, there is a case for at least matching pharmaceutical research budgets with budgets for research on making the dietary remission of type 2 diabetes more effective and sustainable. The challenge will be to ensure equitable access to high-quality, structured, effective and cost-effective lifestyle programmes and to cheaper, efficacious and better-tolerated medications to treat overweight and obesity and to prevent or ‘cure’ type 2 diabetes. Involving people living with diabetes and their families and carers in determining management strategies, as well as payers and policymakers, will be key.

### Supplementary Information

Below is the link to the electronic supplementary material.

Supplementary file1 (PPTX 216 KB)

## Abbreviations

DPP: Diabetes Prevention Program
GLP-1: Glucagon-like peptide-1
SGLT2: Sodium–glucose cotransporter-2
