# Supplementary material for: Remission of type 2 diabetes: always more questions, but enough answers for action
Source: Diabetologia. 2024 Jan 8;67(4):602–10. doi: 10.1007/s00125-023-06069-1 (PMC10904507; doi:10.1007/s00125-023-06069-1)
Supplement: Supplementary file 1 — Supplementary file1 (PPTX 216 KB) [file 125_2023_6069_MOESM1_ESM.pptx]

## Slide 1
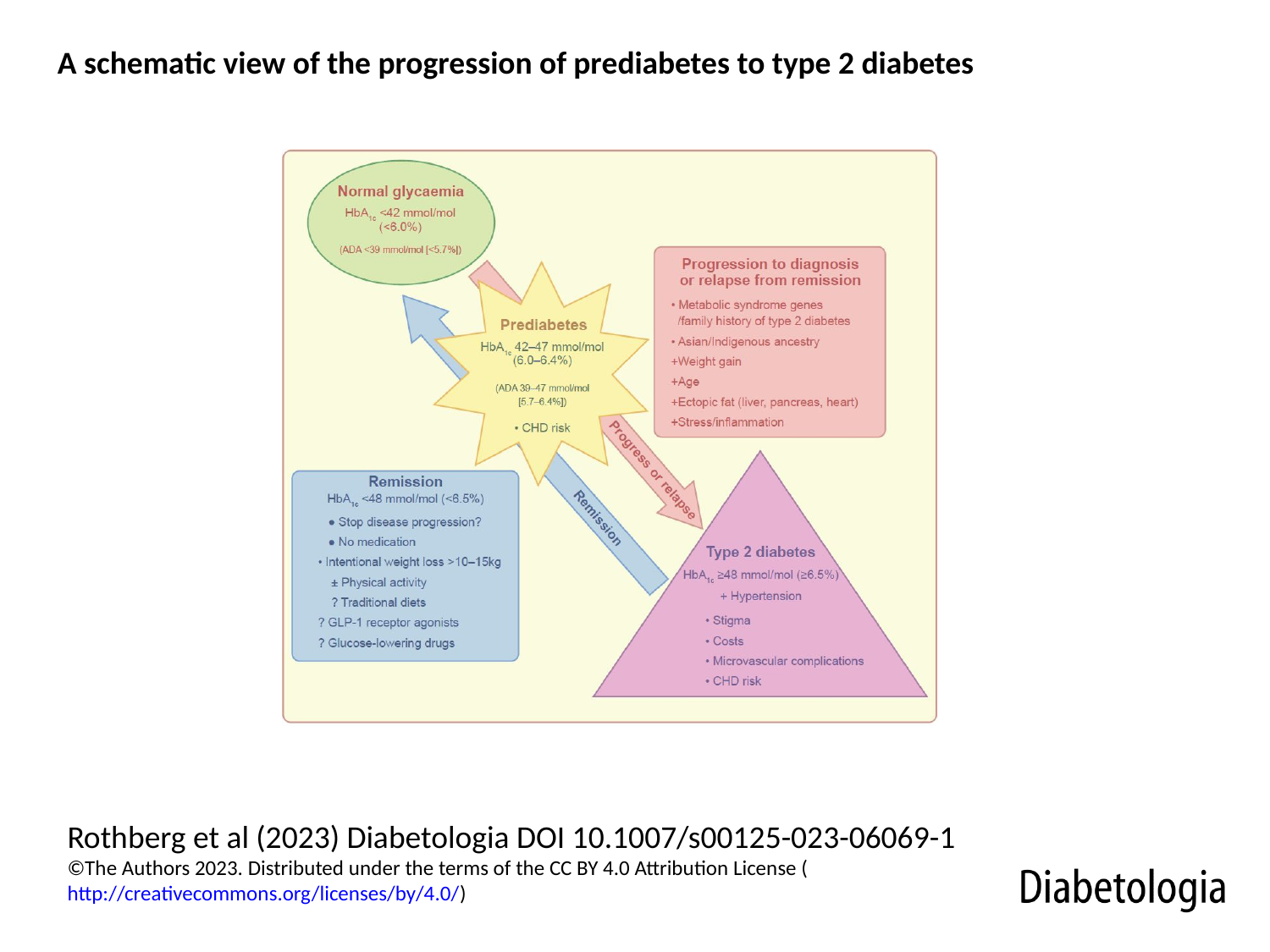

A schematic view of the progression of prediabetes to type 2 diabetes
Rothberg et al (2023) Diabetologia DOI 10.1007/s00125-023-06069-1
©The Authors 2023. Distributed under the terms of the CC BY 4.0 Attribution License (http://creativecommons.org/licenses/by/4.0/)
